# Association between metabolic syndrome and the development of non-alcoholic fatty liver disease

**DOI:** 10.3892/etm.2013.1090

**Published:** 2013-04-29

**Authors:** YI WANG, YU YUAN LI, YU QIANG NIE, YONG JIAN ZHOU, CHUANG YU CAO, LIN XU

**Affiliations:** 1 Departments of Infectious Disease, Guangzhou Medical University, Guangzhou, Guangdong 510180;; 2 Gastroenterology and Hepatology, Guangzhou Institute of Clinical Medicine, Guangzhou First People’s Hospital, Guangzhou Medical University, Guangzhou, Guangdong 510180;; 3 Department of Community Medicine, School of Public Health, University of Hong Kong, Hong Kong, SAR, P.R. China

**Keywords:** non-alcoholic fatty liver disease, metabolic syndrome, elderly individuals, prospective study

## Abstract

The aim of this study was to examine the effects of metabolic syndrome (MS) and the number of MS components on the development of non-alcoholic fatty liver disease (NAFLD). A total of 1,343 males and 574 females aged ≥50 years without NAFLD at baseline were included. Information on lifestyle, including alcohol use and personal history, was collected by face-to-face interviews. Biochemical parameters were assayed using fasting blood samples. NAFLD was diagnosed by abdominal ultrasonography. During follow-up at an average of 4.8 years, 223 patients developed NAFLD. Following adjustment for multiple covariates, age was an independent protective predictor [hazard ratio (HR), 0.96; 95% confidence interval (CI), 0.95–0.98], while the independent risk predictors were obesity (HR, 2.81; 95% CI, 2.14–3.69), higher triglycerides (HR, 2.56; 95% CI, 1.95–3.32) and alanine aminotransferase (HR, 1.004; 95% CI, 1.000–1.008). Participants with a diagnosis of MS had a significantly increased risk of developing NAFLD (HR, 3.17; 95% CI, 2.42–4.14). A greater number of MS components was significantly associated with a higher risk of NAFLD (all adjusted P for trend <0.001). Compared with those without any components of MS, participants with only one component of MS had a 3.6-fold higher risk of developing NAFLD (adjusted HR, 3.64; 95% CI, 1.50–8.88). The diagnosis and the number of components of MS were prospectively associated with the risk of developing NAFLD. Even in those with only one component of MS, the risk increased by 2.6-fold compared with that for the individuals without any components, suggesting a beneficial effect of intervention at the very early stage of MS on the prevention of NAFLD.

## Introduction

Non-alcoholic fatty liver disease (NAFLD) is one of the most common causes of liver-related mortality worldwide (1). NAFLD encompasses a morphological spectrum from simple fatty liver (SFL), non-alcoholic steatohepatitis (NASH) to hepatic cirrhosis. SFL generally has a benign prognosis. It may progress to NASH; however, it seldom progresses to cirrhosis and hepatocellular carcinoma (HCC) (2,3). The prevalence of NAFLD is 20–30% in Western populations (4,5) and 11–15% in Chinese populations (6,7). Due to the westernization of diet and lifestyle, including high fat and sugar and low fiber (8), as well as population aging, the prevalence of NAFLD has been increasing in China (9–11).

NAFLD has been identified as the hepatic manifestation of metabolic syndrome (MS) (12) and the association between NAFLD and MS has been reported in earlier observational studies (13–15). There are a number of common mechanisms between the development of NAFLD and MS. For example, they may have the same pathophysiological basis of insulin resistance (16). A systematic review suggested a potential predictive effect of liver fat on the presence of MS (16). However, no evidence was provided regarding the causal association between NAFLD and MS. The majority of previous studies on the association between MS and NAFLD were based on a cross-sectional design (15,17–22); thus, the temporal sequence was unclear. We identified only one prospective study, involving 3,147 Japanese adults without NAFLD at baseline, in which the presence of MS was associated with the development of NAFLD, after following up for one year (13). Despite the short follow-up period, this study identified that participants with a presence of MS had a 4–11-fold higher risk of NAFLD. Prospective cohort studies on the association of MS and NAFLD in a Chinese population are scarce. We identified only one Chinese study on 117 patients with NAFLD demonstrating that the presence of MS is a significant predictor of NAFLD progression (23). However, no data on the development of NAFLD were presented in the study.

In addition, as the development of MS is a progressive process, which is not simply classified as absent or present, the use of a dichotomous diagnosis may, to a certain extent, limit the predictive ability. The Guangzhou Biobank Cohort Study (GBCS) demonstrated that compared with the presence of MS, the number of MS components is a better predictor of subclinical atherosclerosis (24). The authors also demonstrated that even in participants with only one component of MS, the risk of atherosclerosis was significantly increased compared with that for individuals without any MS components [odds ratio (OR), 2.6] and the risk increased with the increasing number of MS components. However, to the best of our knowledge, the dose-response association between the number of MS components and NAFLD was not examined. The present study is the first large prospective cohort study evaluating the effect of MS, incorporating the presence of MS and the number of MS components, on the development of NAFLD. We hypothesized that there is a dose-response relationship between the number of MS components and the development of NAFLD, with those having one component of MS presenting a higher risk of developing NAFLD.

## Subjects and methods

### Study participants

A total of 3,509 individuals aged ≥50 years receiving an annual physical check-up in the First Municipal People’s Hospital of Guangzhou, China were recruited to the study from April 2005 to December 2005. The majority of the participants were employees of various companies or organizations in Guangzhou. All participants were permanent residents of Guangzhou and were expected to have repeated examinations annually or biennially, which made the follow-up easier. All participants provided written informed consent prior to participation. The study was approved by the Medical Ethics Committee of the First Municipal People’s Hospital.

### Data collection and measurements

At baseline, all participants were asked to come in the morning after fasting for >10 h. Fasting blood samples were collected for measuring conventional risk factors of liver or cardiovascular disease (CVD), including lipids, glucose, alanine aminotransferase (ALT), uric acid (UA) and inflammatory markers. Face-to-face interviews and physical examinations were performed by well-trained nurses or physicians. Demographic and lifestyle information was collected by a standardized questionnaire. Subjects were asked to recall the amount of time during the past week spent on leisure-time exercise (23). Exercise was categorized into three groups according to the frequency per week and average amount of time spent per occasion: regular (≥3 times per week and >20 min each time), seldom (≤1 times per week) and moderate (between seldom and regular). Alcohol consumption was classified into never, occasional, moderate and excessive based on the usual frequency of intake and the usual amount per occasion. Those who did not drink any alcohol throughout their life were classified as non-drinkers. Those who drank <1 occasion per week or drank only on special occasions in the past one year were classified as occasional drinkers. Moderate drinkers were regular drinkers (≥1 occasion per week) who drank <20 g alcohol per day in males or <10 g alcohol per day in females, while excessive drinkers were regular drinkers with alcohol consumption of ≥20 g per day in males or ≥10 g per day in females.

### Diagnostic criteria of NAFLD and MS

Following exclusion of subjects with excessive alcohol consumption and viral or auto-immune liver disease, NAFLD was diagnosed by abdominal ultrasound, which is a widely accessible imaging technique with high diagnostic accuracy and reliability for the detection of fatty liver (25,26). An ultrasonographic examination was performed by an experienced radiologist using a real-time scanner (3.5 MHz; Siemens, Adama, German) equipped with a convex-array probe. The radiologist who performed the ultrasonographic examinations was blinded to the research programs. The ultrasonographic patterns of NAFLD were as follows: i) Bright liver or hepatorenal echo contrast: a ‘bright liver’ was determined when high-level intensive echoes arose from the hepatic parenchyma. The diagnosis of ‘hepatorenal echo contrast’ was based on evident ultrasonographic contrast between the hepatic and right renal parenchyma of the right intercostal sonogram in the mid-axillary line; ii) blurring of the intrahepatic bile ducts; iii) increase of liver volumes and blunt liver edges; iv) obscuring of the hepatic vein trunk; and v) deep attenuation: attenuation of the echo level in the deep region. The presence of NAFLD was diagnosed as the presence of item i) plus any one of items ii–v). Evaluation of the results of ultrasonography was performed by a radiologist, with the results being double-checked by another experienced radiologist to ensure unbiased evaluation.

According to a modified definition from the most updated joint statement (27), participants were defined as having MS if the patient had three or more of the following conditions: i) body mass index (BMI) >25 kg/m^2^; ii) elevated triglycerides (TG; ≥1.7 mmol/l) or specific treatment for this lipid abnormality; iii) reduced high-density lipoprotein (HDL)-cholesterol, (<1.03 mmol/l in males and <1.30 mmol/l in females) or specific treatment for this lipid abnormality; iv) elevated blood pressure [systolic blood pressure (SBP) ≥130 mmHg or diastolic blood pressure (DBP) ≥85 mmHg] or treatment of previously diagnosed hypertension; and v) elevated fasting plasma glucose (FPG; ≥5.6 mmol/l) or previously diagnosed type 2 diabetes. In addition, the number of MS components was also calculated and included in the data analysis.

Follow-up examination was performed from June 2010 to October 2010 (mean follow-up period, 4.8 years; standard deviation, 0.44). A total of 815 participants were excluded due to viral hepatitis and other liver diseases, or for being regular excessive drinkers. As our subjects were elderly Chinese individuals aged ≥50 years, the majority of the subjects included in the present analysis were non-drinkers or occasional drinkers (93.1%). Moreover, 282 participants not returning for follow-up or with missing data on important variables, including components of MS or ultrasound imaging, and 495 participants with NAFLD at baseline examination were also excluded from the present data analysis. Finally, 1,917 participants (1,343 males and 574 females) without NAFLD at baseline and with all variables of interest were included in the present data analysis.

### Statistical analysis

All data analysis was performed using SPSS 17.0 for Windows (SPSS, Inc., Chicago, IL, USA). Analysis of variance (ANOVA) was used to examine differences for continuous variables and χ^2^ was used for categorical variables, with adjustment of age and gender to control for the potential confounding effect of covariates. Cox proportional hazards regression models were used to assess the association between MS or the number of MS components and the development of NAFLD. Risk or protective factors that are associated with the development of NAFLD in the univariate model were considered potential confounders and were included in the final Cox proportional model. The P-value for trend was calculated by including the number of MS components as a continuous variable in the model. Different adjustment models were used to determine the independent correlation between NAFLD and MS or its components.

## Results

Of the 1,917 participants (1,343 males and 574 females) without NAFLD at baseline, 223 developed NAFLD during an average follow-up of 4.8 years [95% confidence interval (CI), 4.2–5.1]. The incidence of NAFLD was 25.0 per 1,000 person-years (95% CI, 21.9–28.5); 24.6 per 1,000 person-years (95% CI, 21.0–28.8) in males and 25.9 per 1,000 person-years (95% CI, 20.5–32.9) in females.

Table I shows that the number of participants with 0, 1, 2, 3, 4 and 5 components of MS were 282, 610, 534, 337, 129 and 25, respectively. The number of MS components was positively associated with older age, CVD history, BMI, TG, FPG, UA, SBP and DBP (all P<0.001), and negatively associated with education level and HDL-cholesterol (both P<0.001). No association between the number of MS components and gender, exercise, total and LDL-cholesterol, ALT and blood urea nitrogen (BUN) was identified (Table I).

Table II shows that in the participants who did not have NAFLD at baseline, the significant protective predictors for the development of NAFLD are younger age [hazard ratio (HR), 0.97; 95% CI, 0.96–0.99] and HDL-cholesterol (HR, 0.34; 95% CI, 0.22–0.52), whereas the significant risk factors are personal CVD history (HR, 1.66; 95% CI, 1.21–2.28), BMI (HR, 1.25; 95% CI, 1.20–1.30), TG (HR, 1.36; 95% CI, 1.27–1.46), ALT (HR, 1.003; 95% CI, 1.001–1.006), UA (HR, 1.002; 95% CI, 1.001–1.003), SBP (HR, 1.008; 95% CI, 1.001–1.015) and DBP (HR, 1.03; 95% CI, 1.01–1.04).

Table III shows that, following mutual adjustment in the multivariate Cox model, the independent and protective predictor is age (HR, 0.97; 95% CI, 0.95–0.98), while the independent risk predictors are obesity (HR, 2.81; 95% CI, 2.14–3.69), higher TG (HR, 2.56; 95% CI, 1.95–3.32) and ALT (HR, 1.004; 95% CI, 1.000–1.008).

Table IV shows that among those without NAFLD at baseline, the HR was significantly increased in those with the diagnosis of MS (HR, 3.17; 95% CI, 2.42–4.14) following adjustment for multiple potential confounders. An increasing number of MS components was significantly associated with risk of NAFLD (all P for trend <0.001). Participants with only one component of MS had a 3.6-fold higher risk of developing NAFLD compared with those without any components of MS (adjusted HR, 3.64; 95% CI, 1.50–8.88).

Table V shows that compared with participants without MS at baseline and follow-up, participants with MS at baseline or at follow-up only, or at baseline and follow-up were significantly associated with an increased risk of developing NAFLD [adjusted OR, 2.21; 95% CI, 1.49–3.27; 1.65 (1.03–2.65) and 4.69 (3.42–6.42), respectively].

## Discussion

To the best of our knowledge, the present large-scale prospective study is the first to show that the number of MS components significantly predicts the development of NAFLD in elderly Chinese individuals without NAFLD at baseline. We identified that even in participants with only one component of MS, the risk of NAFLD was increased by >2.6 fold, suggesting that the presence of a greater number of risk factors is more important than the actual diagnosis of MS in predicting the risk of NAFLD. Moreover, in individuals with MS at baseline and follow-up, the risk of developing NAFLD was 4.7-fold higher compared with that in individuals without MS and was >2-fold higher compared with that in individuals with MS at baseline or at follow-up only.

Although a large body of evidence was identified in the literature regarding the cross-sectional association between metabolic disorders and NAFLD (15,17–22,28), the longitudinal effect of MS on the development of NAFLD was unclear. An earlier survey in Shanghai, China including 3,175 middle-aged adults, identified that participants with metabolic disorders, including central obesity, diabetes, dyslipidemia or hypertension, increased the risk of fatty liver by 33-, 32-, 23- or 23-fold, respectively (19). The risk of fatty liver due to MS was increased by ∼39-fold, which was much higher compared with findings from other studies. However, interpretation of results from the study should be cautious since the study did not control for any other well-known confounding factors, including age, gender, alcohol intake or ALT (16,29), which may lead to overestimation of MS effects. Moreover, since information on alcohol consumption was not collected in the study, participants with fatty liver were not classified as having alcohol-induced fatty liver or non-alcoholic fatty liver. Another cross-sectional study on 876 Taiwanese adults demonstrated a much smaller OR of NAFLD for higher TG, hyperglycemia, central obesity or the presence of MS compared with the Shanghai study (all ORs were ∼2.2–2.4) following adjustment for potential confounders, including age, gender, ALT and UA (22). In a US study of 1,323 adolescents, using ALT >40 U/l as a proxy of NAFLD, subjects with MS were at an ∼11-fold higher risk of NAFLD following adjustment for multiple cofounders (20). The authors identified significant modification effects of gender or ethnicity with MS on NAFLD, with the risk of NAFLD being higher in males and in non-Hispanics compared with that in females and Hispanics, suggesting important roles of genetic, biological or environmental modifiers in the development of NAFLD. In the present study, we did not observe a significant interaction between gender and MS for the development of NAFLD, which is in line with the majority of the previous studies (16,21). Due to the homogeneity of our study sample, the modification effect of ethnicity was not assessed. Further large studies, including different ethnic groups are warranted to clarify the effect. The major limitation of the US study in adolescents was the use of ALT as a proxy of NAFLD; therefore, misclassification of the NAFLD was likely. A more recent cross-sectional study of 2,394 middle-aged Chinese individuals demonstrated a much higher prevalence of NAFLD in participants with MS than in those without MS (56–70% vs. 13–15%) (18). However, the study was more of a descriptive study, rather than an analytical study and no risk prediction model was used to evaluate the association between MS and NAFLD. Confounding effects from other risk factors, including age and gender, were not controlled. In addition, the cross-sectional association of MS with NAFLD was also reported in patients with pre-diabetes or diabetes in an Korean study of 1,365 subjects (15). The authors identified that MS according to the International Diabetes Federation (IDF) definition had the highest ORs of NAFLD compared with other definitions of MS, including those of the World Health Organization or the Adult Treatment Panel III of the National Cholesterol Education Program. As the temporal sequence between MS and NAFLD was unclear, the authors also recommended further studies to determine whether NAFLD is a long-term prognostic factor of MS. In the present study, we used the most updated definition of MS from a Joint Interim Statement of six organizations of the world (27) and examined the effect of MS on NAFLD after excluding those with NAFLD at baseline to avoid the problem of reverse causality. We consider that results from the present study are likely to provide important and supplementary information for clinical and public health practice in reducing or preventing the development of liver-related disease.

Moreover, as the causal correlation between MS and NAFLD was unclear, it may be two-way. Increased fat accumulation in the liver may suppress hepatic insulin clearance and lead to hyperinsulinemia or hyperglycemia (30); this effect is independent of obesity (31). Several earlier studies demonstrated a higher risk of metabolic disorders in patients with NAFLD than in those without (17,21). However, since data on metabolic disorders and NAFLD were collected and analyzed cross-sectionally, the causal association between metabolic disorders and NAFLD is unclear. It is likely that ectopic fat storage in the muscle, liver or pancreatic β cells, which has been reported to be a major cause of MS, may in turn cause insulin secretory defects, hepatic and peripheral insulin resistance and hepatic steatosis (32). Prospective studies examining the effect of MS on NAFLD are scarce, with only one Japanese study demonstrating that the presence of MS at baseline is a significant predictor of the development of NAFLD after following-up for approximately one year (13). However, the dose-response effects of the number of MS components on NAFLD was not assessed, possibly since the insufficient cases of NAFLD occurring during the relatively short follow-up period were not able to support a dose-response analysis. Hence, the current study, for the first time, demonstrated that an increasing number of MS components significantly predicted the development of NAFLD 4.8 years later. We also identified that compared with the risk of NAFLD in those without any components of MS, the risk was significantly increased by 3.6-fold in those with only one component of MS, which was higher than that for the actual diagnosis of MS (HR, 3.17), suggesting that identifying the number of MS components may be more important than the diagnosis of MS in predicting NAFLD.

There are several limitations that should be considered. Firstly, ultrasonography may lead to an incorrect diagnosis of NAFLD and may not distinguish steatohepatitis from simple steatosis. The present study was limited by lacking histological data to support the diagnosis of NAFLD. Liver biopsy is required for definitive diagnosis of NAFLD. However, since liver biopsy is invasive and expensive, it may be a challenge to persuade patients, particularly elderly individuals, to undergo liver biopsy. Moreover, it is hard to use liver biopsy for population-based epidemiological studies. Although computed tomography (CT)/magnetic resonance imaging (MRI) are considered accurate techniques to assess steatosis (33–35), the radiation exposure, as well as the high cost, limit the wide used for fatty liver diagnosis in population-based research or screening programs. According to a recent meta-analysis, ultrasonography has a high reliability and validity in the diagnosis of fatty liver compared with histology, with sensitivity and specificity being 84.8 and 93.6%, respectively (26). Since it is relatively cheap, non-invasive and highly accessible, ultrasound has been suggested to be used for large population- or clinical-based research or screening for fatty liver. Secondly, our study comprised an homogenous sample of elderly Chinese individuals so the modification effects from ethnicity were not assessed. Further studies including different ethnic groups are warranted. Finally, a well-validated simple questionnaire was used to collect data on demographic or lifestyle factors. Detailed information on certain potential risk factors of fatty liver, including diet, were not assessed. For example, data on the total daily energy intake, frequency and amount of meat or vegetable consumption were not available in the present study. Whether diet plays a role in the association between MS and NAFLD is unclear. Further studies including more detailed assessment of diet or total energy intake are required.

In conclusion, we identified that the number of MS components was more useful than the presence of MS in predicting NAFLD. Individuals with only one component of MS had a 3.6-fold higher risk of developing NAFLD, suggesting a significantly higher risk of NAFLD in the extremely early stage of MS. The present results have important public health implications. Early screening and treatment for any components of MS may be useful for preventing the development of NAFLD.

## Figures and Tables

**Table I.** Age- and gender-adjusted clinical and biochemical parameters by number of MS components in 1,917 participants without NAFLD at baseline.

Parameter	Number of components						P-value	P for trend
	0 (n=282)	1 (n=610)	2 (n=534)	3 (n=337)	4 (n=129)	5 (n=25)		
Age (years)^a^	68.3 (67.5–69.2)	69.4 (68.8–70.0)	70.5 (69.9–71.1)	69.7 (68.9–70.5)	70.6 (69.3–71.9)	72.8 (69.9–75.7)	<0.001	<0.001
Male gender (%)	65.96	72.62	71.16	63.76	79.84	64	0.14	0.66
Education (%)								
College or above	74.30	68.10	65.30	61.90	51.20	44.0	<0.001	<0.001
Middle school	23.60	30.10	31.30	33.10	43.40	48.0		
Primary or below	2.10	1.80	3.40	5.00	5.40	8.0		
Exercise (%)								
Regular	62.30	55.70	49.60	50.20	51.20	53.2	0.74	0.41
Moderate	18.20	23.20	33.20	30.20	29.40	30.8		
Seldom	19.50	21.10	17.20	19.60	19.40	16.0		
Personal CVD history (% yes)	42.55	63.28	76.59	75.96	91.47	88	<0.001	<0.001
BMI (kg/m^2^)^a^	21.50 (21.19–21.81)	22.58 (22.37–22.78)	23.75 (23.53–23.97)	25.39 (25.12–25.67)	26.34 (25.88–26.81)	27.17 (26.03–28.31)	<0.001	<0.001
TC (mmol/l)^a^	5.50 (5.37–5.62)	5.50 (5.41–5.58)	5.58 (5.49–5.67)	5.63 (5.52–5.74)	5.48 (5.29–5.69)	5.53 (5.07–5.99)	0.42	0.15
HDL-C (mmol/l)^a^	1.62 (1.58–1.65)	1.53 (1.50–1.56)	1.40 (1.38–1.43)	1.33 (1.29–1.36)	1.21 (1.15–1.27)	1.09 (0.95–1.24)	<0.001	<0.001
LDL-C (mmol/l)^a^	3.03 (2.95–3.12)	3.00 (2.94–3.05)	3.10 (3.03–3.16)	3.11 (3.03–3.20)	3.04 (2.91–3.17)	3.23 (2.90–3.55)	0.10	0.08
TG (mmol/l)^a^	0.98 (0.89–1.07)	1.10 (1.04–1.16)	1.37 (1.30–1.43)	1.72 (1.64–1.81)	2.25 (2.11–2.39)	2.66 (2.32–2.99)	<0.001	<0.001
FPG (mmol/l)^a^	4.96 (4.83–5.09)	5.25 (5.16–5.34)	5.76 (5.66–5.85)	6.08 (5.96–6.20)	6.48 (6.29–6.68)	6.43 (5.95–6.91)	<0.001	<0.001
ALT (U/l)^a^	23.47 (21.37–25.56)	22.27 (20.86–23.67)	22.83 (21.33–24.34)	23.68 (21.79–25.58)	26.10 (22.95–29.25)	23.56 (15.80–31.32)	0.36	0.25
BUN (mmol/l)^a^	5.99 (5.81–6.17)	6.06 (5.94–6.18)	6.11 (5.98–6.24)	5.99 (5.83–6.16)	6.27 (5.99–6.54)	5.76 (5.49–6.43)	0.43	0.52
UA (mmol/l)^a^	325 (313–338)	352 (344–360)	367 (358–376)	377 (366–389)	383 (365–402)	417 (371–462)	<0.001	<0.001
SBP (mmHg)^a^	115 (113–117)	132 (131–133)	138 (137–139)	141 (139–143)	142 (138–145)	145 (139–152)	<0.001	<0.001
DBP (mmHg)^a^	68 (67–69)	75 (74–75)	77 (76–77)	79 (78–81)	80 (79–81)	81 (76–85)	<0.001	<0.001

MS, metabolic syndrome; NAFLD, non-alcoholic fatty liver disease; CVD, cardiovascular disease; BMI, body mass index; TC, total cholesterol; HDL-C, high-density lipoprotein cholesterol; LDL-C, low-density lipoprotein cholesterol; TG, triglycerides; FPG, fasting plasma glucose; ALT, alanine aminotransferase; BUN, blood urea nitrogen; UA, uric acid; SBP, systolic blood pressure; DBP, diastolic blood pressure.

a 95% confidence intervals in parentheses.

**Table II.** Univariate Cox proportional hazards models for the development of NAFLD in 1,917 participants without NAFLD at baseline.

Parameter	Hazard ratio	95% Confidence interval	P-value
Age (years)	0.97	0.96–0.99	0.002
Male gender	0.96	0.72–1.28	0.79
Education			
Primary or below	1.00		
Middle school	1.59	0.64–3.94	0.35
College or above	1.53	0.63–3.72	0.32
Exercise			
Seldom	1.00		
Moderate	0.70	0.17–2.87	0.62
Regular	0.79	0.58–1.09	0.15
Personal CVD history			
No	1.00		
Yes	1.66	1.21–2.28	0.002
BMI (kg/m^2^)	1.25	1.20–1.30	<0.001
TC (mmol/l)	1.007	0.89–1.14	0.91
HDL-C (mmol/l)	0.34	0.22–0.52	<0.001
LDL-C (mmol/l)	1.15	0.97–1.37	0.11
TG (mmol/l)	1.36	1.27–1.46	<0.001
FPG (mmol/l)	1.09	0.99–1.19	0.07
ALT (U/l)	1.003	1.001–1.006	0.02
BUN (mmol/l)	0.96	0.88–1.05	0.40
UA (mmol/l)	1.002	1.001–1.003	<0.001
SBP (mmHg)	1.008	1.001–1.015	0.03
DBP (mmHg)	1.03	1.01–1.04	<0.001

NAFLD, non-alcoholic fatty liver disease; CVD, cardiovascular disease; TC, total cholesterol; HDL-C, high-density lipoprotein cholesterol; LDL-C, low-density lipoprotein cholesterol; TG, triglycerides; ALT, alanine aminotransferase; FPG, fasting plasma glucose; UA, uric acid; BUN, blood urea nitrogen; BMI, body mass index; SBP, systolic blood pressure; DBP, diastolic blood pressure.

**Table III.** Hazard ratios (95% confidence interval) for NAFLD by MS components and other selected factors in 1,917 participants without NAFLD at baseline.

Parameter	Number (%) with NAFLD	Crude HRs (95% CI)	Adjusted HRs (95% CI)^b^
Age	-	0.97 (0.96–0.99)^d^	0.96 (0.95–0.98)^e^
Personal CVD history			
No	49 (8.25)	1.00	
Yes	173 (13.27)	1.66 (1.21–2.28)^c^	1.35 (0.96–1.89)
Obesity^a^	124 (22.32)	3.20 (2.54–4.31)^e^	2.81 (2.14–3.69)^e^
Elevated triglycerides^a^	100 (23.09)	3.02 (2.28–3.87)^e^	2.56 (1.95–3.32)^e^
Low HDL-C^a^	46 (16.67)	1.51 (1.09–2.09)^c^	1.20 (0.86–1.65)
Elevated blood pressure^a^	161 (13.15)	1.52 (1.13–2.05)^d^	1.28 (0.95–1.74)
Elevated fasting glucose^a^	104 (14.75)	1.53 (1.17–1.99)^d^	1.27 (0.97–1.66)
ALT	-	1.003 (1.001–1.006)^c^	1.004 (1.000–1.008)^c^
UA	-	1.002 (1.001–1.003)^e^	1.001 (1.000–1.002)

a Obesity defined as BMI >25 kg/m^2^ in males and females; elevated triglycerides, plasma triglyceride ≥1.7 mmol/l or specific treatment for this abnormality; low HDL-C, plasma HDL-C ≤1.03 mmol/l in males and ≤1.30 mmol/l in females; high blood pressure, systolic blood pressure ≥130 mmHg or diastolic blood pressure ≥85 mmHg or treatment of previously diagnosed hypertension; high fasting glucose, fasting plasma glucose ≥5.6 mmol/l.

b Risk factors in the table above were mutually adjusted.

c P<0.05 compared with group without the risk factor;

d P<0.01 compared with group without the risk factor;

e P<0.001 compared with group without the risk factor. NAFLD, non-alcoholic fatty liver disease; MS, metabolic syndrome; HR, hazard ratio; CI, confidence interval; CVD, cardiovascular disease; HDL-C, high-density lipoprotein cholesterol; ALT, alanine aminotransferase; UA, uric acid; BMI, body mass index.

**Table IV.** Cox proportional hazards regression model (hazard ratio, 95% confidence interval) on the association between MS at baseline and the development of NAFLD in 1,917 participants without NAFLD at baseline.

Variable	Presence of MS		Number of MS components				P for trend
	No	Yes	0	1	2	3+	
Total (n=1917)	1426	491	282	610	534	491	
NAFLD (n=223)	105 (7.4)	118 (24.0)	6 (2.1)	46 (7.5)	54 (10.1)	117 (24.0)	<0.001
Crude HR	1.00	3.48 (2.67–4.52)	1.00	3.51 (1.50–8.22)^a^	4.91 (2.11–11.42)^b^	12.26 (5.40–27.85)^b^	<0.001
Model 1	1.00	3.29 (2.52–4.30)	1.00	3.43 (1.46–8.07)^a^	4.85 (2.07–11.36)^b^	11.65 (5.08–26.70)^b^	<0.001
Model 2	1.00	3.17 (2.42–4.14)	1.00	3.64 (1.50–8.88)^a^	5.04 (2.08–12.25)^b^	11.82 (4.97–28.11)^b^	<0.001

Model 1, adjusting for age and personal cardiovascular disease (CVD) history. Model 2, additionally adjusting for alanine aminotransferase (ALT) and uric acid.

a P<0.01 compared with no components of MS;

b P<0.001 compared with no components of MS. NAFLD, non-alcoholic fatty liver disease; MS, metabolic syndrome; HR, hazard ratio.

**Table V.** Change of MS status and the development of NAFLD at follow-up; odds ratio (95% confidence interval).

Variable	Change of MS status			
	No MS	MS at baseline only	MS at follow-up only	MS at baseline and follow-up
Total no.	1,239	236	187	255
NAFLD, n (%)	83 (6.7)	36 (15.3)	22 (11.8)	82 (32.2)
Crude OR	1.00	2.42 (1.65–3.57)^b^	1.82 (1.14–2.90)^a^	5.24 (3.85–7.12)^b^
Model 1	1.00	2.30 (1.56–3.40)^b^	1.70 (1.06–2.73)^a^	4.91 (3.59–6.71)^b^
Model 2	1.00	2.21 (1.49–3.27)^b^	1.65 (1.03–2.65)^a^	4.69 (3.42–6.42)^b^

Model 1, adjusting for age and CVD history. Model 2, additionally adjusting for ALT and uric acid.

a P<0.01 compared with no components of MS;

b P<0.001 compared with no components of MS. MS, metabolic syndrome; NAFLD, non-alcoholic fatty liver disease; OR, odds ratio.
